# Soft Lithography and Minimally Human Invasive Technique for Rapid Screening of Oral Biofilm Formation on New Microfabricated Dental Material Surfaces

**DOI:** 10.1155/2018/4219625

**Published:** 2018-01-14

**Authors:** Marta Alvarez-Escobar, Sidónio C. Freitas, Derek Hansford, Fernando J. Monteiro, Alejandro Pelaez-Vargas

**Affiliations:** ^1^Faculty of Dentistry, Universidad Cooperativa de Colombia, Medellín, Colombia; ^2^Department of Biomedical Engineering, The Ohio State University, Columbus, OH 43210, USA; ^3^Instituto de Investigação e Inovação em Saúde (i3S), Instituto de Engenharia Biomédica (INEB) and DMME, Faculdade de Engenharia, Universidade do Porto (FEUP), Porto, Portugal

## Abstract

**Introduction:**

Microfabrication offers opportunities to study surface concepts focused to reduce bacterial adhesion on implants using human minimally invasive rapid screening (hMIRS). Wide information is available about cell/biomaterial interactions using eukaryotic and prokaryotic cells on surfaces of dental materials with different topographies, but studies using human being are still limited.

**Objective:**

To evaluate a synergy of microfabrication and hMIRS to study the bacterial adhesion on micropatterned surfaces for dental materials.

**Materials and Methods:**

Micropatterned and flat surfaces on biomedical PDMS disks were produced by soft lithography. The hMIRS approach was used to evaluate the total oral bacterial adhesion on PDMS surfaces placed in the oral cavity of five volunteers (the study was approved by the University Ethical Committee). After 24 h, the disks were analyzed using MTT assay and light microscopy.

**Results:**

In the present pilot study, microwell structures were microfabricated on the PDMS surface via soft lithography with a spacing of 5 *µ*m. Overall, bacterial adhesion did not significantly differ between the flat and micropatterned surfaces. However, individual analysis of two subjects showed greater bacterial adhesion on the micropatterned surfaces than on the flat surfaces.

**Significance:**

Microfabrication and hMIRS might be implemented to study the cell/biomaterial interactions for dental materials.

## 1. Introduction

The formation of oral biofilms on natural materials (e.g., enamel and dentin) and restorative biomaterials promotes the development of diseases, such as dental caries, periodontitis, and peri-implantitis [1, 2]. Microbial adhesion is the first step in colonization and the formation of a biofilm, in which microorganisms and extracellular material accumulate on a solid surface [3]. Biofilms have been defined as communities of microorganisms that grow embedded in a matrix of exopolysaccharides that affect inert surfaces or living tissues [4], where such formation can be produced by any microorganism if suitable environmental conditions are provided.

The microenvironment plays a decisive role in the formation of the oral biofilm on dental materials due to direct or indirect interactions [5]. Factors such as pH, temperature, and saliva, among others, affect biofilm composition [6]. These interactions are a challenge in using in vitro approaches (i.e., bacterial static or dynamic culture reactors and microfluidic devices) due to intra- and interindividual variations in the oral environment through health and disease conditions. In addition, surface properties are one of the major microenvironmental factors that substantially influence biofilm formation. Variations in free surface energy and surface roughness promote plaque formation and maturation [7, 8].

Soft lithography allows the production of random or ordered surfaces in a controlled manner. Such surfaces modulate the cellular response [9], specifically cell adhesion, metabolism, orientation, adhesion, growth, and differentiation in vitro [10–12]. The relationship between microfabricated surfaces and bacterial behaviour show limited information [4, 13–15] and human studies are narrow due to bioethical regulations. The challenge to carry out translational research to surface modificated biomaterials is a intensive, time-consuming and cost labor. Human rapid screening might provide new successful therapies to patients in a short time. Rimondini et al. [6] introduced a minimally invasive technique to evaluate the adhesion of bacteria and biofilms in dental materials with random topographies generated by chemical or physical processing based on subtractive techniques. However, ordered topographies are hard to produce by traditional processing, but it was solved using microengineered techniques. To the best of our knowledge, a synergy of hMIRS and additive additives such as soft lithography has not been reported to evaluate biofilm formation on dental and implant material surfaces.

The aim of the present study was to evaluate a synergy of microfabrication and hMIRS to study the bacterial adhesion on micropatterned surfaces for dental materials.

## 2. Materials and Methods

This pilot study had approval from the ethical committee of the Universidad Cooperativa de Colombia, Medellín, Colombia. Five subjects between 18 and 35 years of age were invited, who met the following inclusion criteria: no antibiotic treatment during the 3 months prior to the test, no use of orthodontic appliances, nonsmoker, non-dental student, and have signed informed consent.

Microfabricated surfaces were obtained using biomedical PDMS (FDA approved) processed by soft lithography technology, which included the manufacturing of a master model by UV photolithography for two different purposes: (a) to test pattern transferability using raised and recessed features of different sizes, and (b) to fabricate the test geometries (pillar arrays with a 5 *µ*m diameter, 5 *µ*m height, and 5 *µ*m spacing) needed for the biofilm study [16]. A PDMS substrate was fabricated via replica casting, where PDMS (Silastic MDX4-4210, Dow Corning, USA) was mixed with a curing agent at a 10 : 1 ratio and cast on the master to generate a negative replica of the surface. PDMS flat surfaces were fabricated using the same procedure as microfabricated surface, but without the master. Characterization of the micropatterned and flat surfaces was performed using light microscopy (Primo Star, Zeiss, Switzerland) and scanning electron microscopy (Hitachi, S-3000H, Japan).

**Figure 1:**
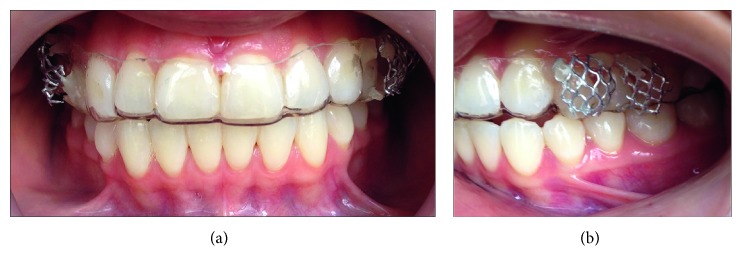
Intraoral modified Essix retainer with metal baskets.

Modified Essix retainers were fabricated with two metal baskets per hemiarch where transparent PDMS disks of 6 mm diameter were placed with flat and micropatterned surfaces (Figure 1). All subjects were instructed to wear the modified Essix retainer for 24 hours, removing it only for eating, brushing, and contact sports.

After the plates were removed from the mouth, the disks were washed three times with saline solution (Baxter, USA). The disks were incubated in a solution of 3-(4,5-dimethylthiazol-2-yl)-2,5-diphenyltetrazolium bromide (MTT, Molecular Probe, USA) at 0.5 mg/ml in saline solution for 2 hours at 37°C. Quantification of the area covered by the biofilm was performed on 6 random micrographs obtained by optical microscopy through an ad hoc digital image-processing strategy that included binarization, edge detection, segmentation, and pixel counting using ImageJ 1.51 g [17, 18].

### 2.1. Statistical Analysis

A comparison of the area covered by biofilm was performed for the two evaluated surfaces with a nonparametric Wilcoxon rank-sum test blocked by the subject using R software.

## 3. Results

**Figure 2:**
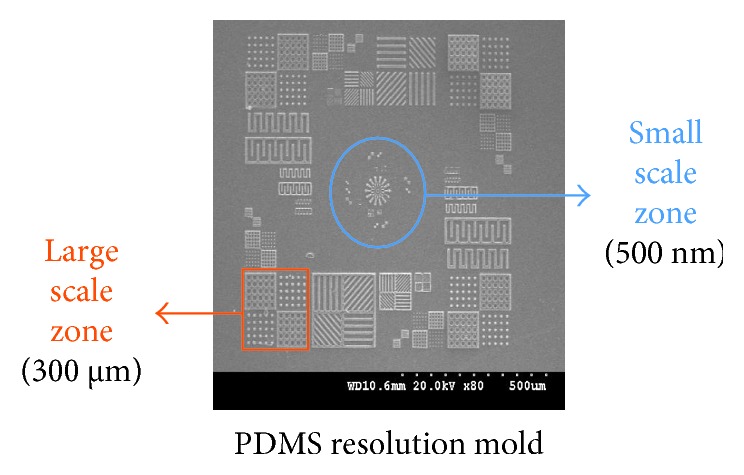
Resolution test. Several geometries with scales (from 500 nm to 300 *µ*m) were transferred from silicon wafer to PDMS.

Figure 2 shows the results of PDMS micropatterned arrays from 500 nm to 300 *µ*m.

**Figure 3:**
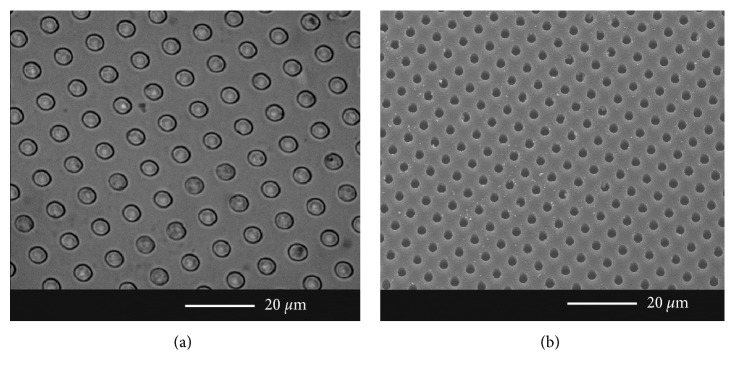
Micropatterned surfaces on PDMS: (a) 100x optical micrograph and (b) scanning electron micrograph.

Figure 3 shows optical and scanning electron micrographs of the micropatterned surfaces on PDMS. A 5 *μ*m well structure (depth, diameter, and spacing) is observed with no defects.

**Figure 4:**
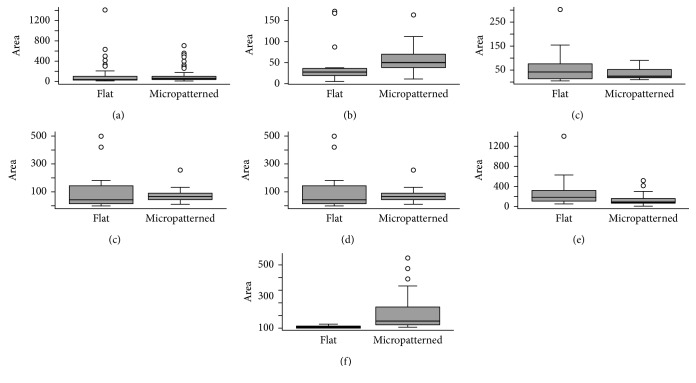
Comparison between micropatterned surfaces and flat surfaces. (a) Overall. (b–f) Subjects 1–5.

Overall (Figure 4), the area covered by the bacteria did not significantly differ between the micropatterned surfaces and the flat surfaces (*p*=0.06). Patient-to-patient comparison between the micropatterned surfaces and the flat surfaces did not show statistically significant differences for subjects 2, 3, and 4 (Figures 4–4). For subjects 1 and 5, significantly less biofilm formation was found on the flat surfaces (Figures 4 and 4).

## 4. Discussion

Bacterial adhesion and biofilm formation on biomaterials is a complex process involving environmental factors, physical and chemical surface properties, and bacterial characteristics [19]. The surface has been extensively studied in terms of free energy and topography and has been associated with bacterial adhesion, growth, and maturation of biofilm [7, 8]. Topography modulates the behavior of eukaryotic cells in terms of adhesion, orientation, growth, differentiation, and apoptosis [20, 21]. Cell adhesion dynamics on ordered microtopographies has been largely studied using in vitro approaches [22, 23], which showed that the response is not universal for all surfaces, materials, and cells. Adherent cells such as rat fibroblasts [22] show a preference for smooth surfaces rather than surfaces with micro/nanopillars. In contrast, glial cells show a positive response for textured surfaces [23].

Andersson et al. [24] studied cell adhesion on smooth and textured surfaces with grooves (15 *μ*m wide and 185 nm deep) and pillars (168 nm diameter and 100 nm high) and found that increasing the height of the topography reduced epithelial cell adhesion. In addition, cells on pillars had a smaller area of covering compared to flat surfaces.

Dalby et al. [25] evaluated a topography of <20 nm that promoted adhesion in a wide range of cell types (endothelial, fibroblast, and mesenchymal). These authors [26] show that small pillar (10 nm) topographies improve the adhesion of fibroblasts compared to 50 nm. Rice et al. [27] also showed that osteoblasts had low adhesion to nanopillars with heights of 160 nm.

In contrast, bacterial studies on micropatterned surfaces are limited. Chung et al. [28] developed micropatterned surfaces by recreating shark skin on PDMS to evaluate the in vitro adhesion of *Staphylococcus aureus* when compared to smooth surfaces. Their results showed that the topography inhibited the development of biofilm. Such response was attributed to the fact that the protruding features of the surfaces provide a physical barrier that prevents the expansion of small colonies of bacteria. Hochbaum and Aizenberg demonstrated bacterial ordering and orientated attachment on the single-cell level induced by nanometer scale periodic surface features [15]. The possible clinical implications of these in vitro findings justify studies based on human models. A systematic review [8] concluded that an increase in the surface roughness greater than Ra 0.2 *μ*m or an increase in the free energy of the surface will facilitate the formation of biofilms in restorative materials.

The present pilot study used a surface model of microwells with a diameter, depth, and interspacing of 5 *μ*m and hMIRS in terms of limited contact duration (24 h) and in contact with uncompromised oral mucosa. General comparisons showed no statistically significant difference in bacterial adhesion between flat and microfabricated surfaces on PDMS. These results might be explained by antiadhesive barrier as a consequence of high hydrophobicity in the biomedical PDMS used, which could be reduced with increased exposure time, but this would be opposite to the rapid screening model described. Modified Essix retainer was used for a period of 24 hours, which matched the in vivo human studies reported in the literature [6, 29, 30]. Auschill et al. [31] evaluated the formation of biofilms over a 120-hour period on different dental materials, including glass and ceramics. They found biofilm formation with thicknesses of 1–17 *μ*m, values that are similar to those reported in 24-hour studies [6, 29, 30].

In the present pilot study, the sample size used was similar to that in in vivo human studies, which had sample sizes ranging from 5 to 10 subjects [6, 29, 30]. These results of the present pilot study found that the synergy of microfabrication by soft lithography and hMIRS might be a powerful tool to evaluate the bacterial/implant-biomaterials interface in human with a minimum risk for subjects. The tested biomedical PDMS model surfaces require further research that includes other factors such as different microstructure features, microscale and nanoscale topographies, and contact duration.
